# Group I PAK Inhibitor IPA-3 Induces Cell Death and Affects Cell Adhesivity to Fibronectin in Human Hematopoietic Cells

**DOI:** 10.1371/journal.pone.0092560

**Published:** 2014-03-24

**Authors:** Kateřina Kuželová, Dana Grebeňová, Aleš Holoubek, Pavla Röselová, Adam Obr

**Affiliations:** Department of Cellular Biochemistry, Institute of Hematology and Blood Transfusion, Prague, Czech Republic; NCMLS, Radboud University Nijmegen Medical Center, Netherlands

## Abstract

P21-activated kinases (PAKs) are involved in the regulation of multiple processes including cell proliferation, adhesion and migration. However, the current knowledge about their function is mainly based on results obtained in adherent cell types. We investigated the effect of group I PAK inhibition using the compound IPA-3 in a variety of human leukemic cell lines (JURL-MK1, MOLM-7, K562, CML-T1, HL-60, Karpas-299, Jurkat, HEL) as well as in primary blood cells. IPA-3 induced cell death with EC50 ranging from 5 to more than 20 μM. Similar range was found for IPA-3-mediated dephosphorylation of a known PAK downstream effector, cofilin. The cell death was associated with caspase-3 activation, PARP cleavage and apoptotic DNA fragmentation. In parallel, 20 μM IPA-3 treatment induced rapid and marked decrease of the cell adhesivity to fibronectin. Per contra, partial reduction of PAK activity using lower dose IPA-3 or siRNA resulted in a slight increase in the cell adhesivity. The changes in the cell adhesivity were also studied using real-time microimpedance measurement and by interference reflection microscopy. Significant differences in the intracellular IPA-3 level among various cell lines were observed indicating that an active mechanism is involved in IPA-3 transport.

## Introduction

Group I p21-activated kinases (PAKs) are implicated in a wide range of cellular processes including cell proliferation, apoptosis, migration and adhesion to the extracellular matrix [1], [2]. PAKs belong to the best known effectors of small GTPases Rac1 and Cdc42 and many of PAK functions are associated with the regulation of cytoskeleton rearrangements. Despite of a high sequence homology, the individual members of group I PAK family (PAK1, PAK2 and PAK3) appear to subserve distinct tasks [1], [3]. While PAK2 expression is ubiquitous, PAK1 is predominantly expressed in brain, muscle and spleen and PAK3 expression is specific for neurons. General knowledge about PAK functions is mainly based on findings obtained using adherent cell models where increased PAK activity usually correlates with increased cell motility and, in the case of solid tumors, higher invasiveness. On the other hand, little is known about the role of PAK kinases in hematopoietic cells [4]. PAK1 or PAK2 expression is upregulated in some types of cancer [5]–[11] and PAKs were suggested to be a suitable target for anti-cancer therapy [2], [11], [12] as well as for the treatment of airway hyperresponsiveness [13] or in conditions of vascular leak [14]. PAK1 was also identified as a major mediator of resistance to phosphoinositide 3-kinase inhibitors in lymphoma cell lines [15]. Attempts to develop a specific small molecule PAK inhibitor resulted in the discovery of IPA-3, an allosteric inhibitor of group I PAK activation [16]–[18] which is suitable for studies of PAK functions although its properties preclude its use in the clinical practice. We have previously reported that IPA-3 treatment of human leukemic JURL-MK1 cells reduced their ability to bind to fibronectin, one of the major components of the bone marrow extracellular matrix [19] and we have also noted IPA-3 toxicity for hematopoietic cells. Very recently, group I PAKs (especially PAK2) were shown to be required for hematopoietic stem cell engraftment, at least in mouse models [20]. In the present work, we thoroughly investigated the effects of PAK inhibition in a panel of human leukemia/lymphoma cell lines as well as in normal primary blood cells.

## Materials and Methods

### Chemicals

IPA-3 was purchased from Sigma-Aldrich (Prague, Czech Republic) and 20 mM stock solution was made in dimethylsulfoxide. To prevent precipitation in water solution, IPA-3 stock solution was diluted 20 fold in 50 mM Tris, pH 8.0, before addition to cell suspension.

Fibronectin fragment (120 kDa cell attachment region) was purchased from Chemicon International (CA, U.S.A.). To prepare fibronectin-coated plate, 50 μl of fibronectin fragment solution (20 μg/ml in distilled water) was added to each well of a Nunc Maxisorp 96-well microtitration plate or of 16-well E-plate for microimpedance measurement and the plates were subsequently incubated overnight in the cold (10°C). Then, the wells were washed three times in PBS and the remaining protein adherence sites were blocked by 200 μl 1% bovine serum albumin (BSA) in PBS for at least 30 min at room temperature. The plate was washed once again in PBS immediately before use.

Antibodies against PAK1 (#2602), PAK2 (#2615), PAK3 (#2609), PAK1/2/3 (#2604) and pPAK2(Ser20) (#2607) were from Cell Signaling, the antibody against pPAK(Ser141/144) was from Abcam (ab5247) or from Cell Signaling (#2606), anti pSer3-cofilin from Sigma (C8992), anti-cofilin (sc-33779) from Santa Cruz, anti-pSer16-stathmin (#3353) from Cell Signaling and anti-stathmin1 (ab11269) from Abcam.

Small interfering RNAs targeting human PAK1 and PAK2 were obtained from Cell Signaling and from Sigma (mission esiRNA, EHU140181 and EHU026721). Control (non-targeting) siRNA was from Santa Cruz (sc-37007).

### Ethics Statement

Samples of normal blood were provided by the authors of the work themselves. Primary leukemic blood cells were isolated from leukapheresis products, following written informed consent of the patient as to the use of biological material for research purposes. The research was approved by the Ethics Committee of the Institute of Hematology and Blood Transfusion.

### Cell Isolation and Culture

JURL-MK1, CML-T1 and Karpas-299 cell lines were purchased from DSMZ (German Collection of Microorganisms and Cell Cultures, Braunschweig, Germany), K562, HL-60 and Jurkat cell lines from the European Collection of Animal Cell Cultures (Salisbury, UK). MOLM-7 and HEL cells were provided by J.Minowada [21] and P.Martin [22], respectively. All cell lines were cultured in RPMI 1640 medium supplemented with 10% fetal calf serum, 100 U/ml penicillin, 100 μg/ml streptomycin at 37°C in 5% CO_2_ humidified atmosphere. The cells were counted using the automated cell counter TC10 (BioRad). As indicated by the counter, the main cell diameter was 12 μm for the majority of cell lines except for CML-T1 cells (8 μm) and K562 and HEL cells (both 16 μm).

Primary blood cells were obtained from the whole blood of healthy donors or from leukapheresis product from patients with chronic or acute myelogenous leukemia. Peripheral blood mononuclear cells (PBMC) were separated by standard density gradient centrifugation using Histopaque-1077 (Sigma) and maintained in RPMI 1640 medium described above.

### Measurement of Cell Metabolic Rate (MTT Assay)

Cells were cultured for 48 h in RPMI 1640 without phenol red and then seeded at 5×10^5^ cells/ml, 100 μl per well, in quadruplicates. IPA-3 at 20 μM concentration or DMSO in Tris buffer (control) were added and the cells were incubated for 2 to 24 h at 37°C. Ten microliters of MTT (5 mg/ml in PBS) were added to each well. After 4 h incubation at 37°C, 100 μl SDS solution (1 g per 10 ml 0.01 M HCl) were added, the cell suspension was thouroughly mixed and the plate was incubated for further 4 h. Thereafter, the absorbance at 590 nm was measured, the blank (RPMI 1640 with MTT, without cells) was subtracted and the cell metabolic rate in IPA-3 treated cells was expressed as relative to the value obtained from the untreated controls.

### Cell Cycle Analysis

For DNA content analysis, the cells (5×10^5^) were harvested, resuspended in 4.5 ml ice cold 70% ethanol, incubated for 30 min at 10°C and stored at −20°C. The day of the analysis, the sample was washed twice in PBS and incubated for 2 h at 10°C in 0.5 ml of modified Vindelov’s propidium iodide buffer (10 mM Tris, pH 8, 1 mM NaCl, 0.1% Triton X-100, 20 μg/ml PI, and 10 K units ribonuclease A). PI fluorescence was measured using LSRFortessa flow cytometer (BD Biosciences).

### Apoptotic DNA Fragmentation (TUNEL*)*

The fraction of cells containing apoptotic DNA breaks was measured by TUNEL assay using the In Situ Cell Death Detection Kit, Fluorescein (Roche Diagnostics GmbH, Mannheim, Germany) following the standard manufacturer’s protocol. Flow cytometry measurements were performed using LSRFortessa flow cytometer (BD Biosciences).

### Caspase-3 Activity Measurement

The *in vitro* caspase-3 activity was determined by fluorometric measurement of the kinetics of 7-amino-4-trifluoromethylcoumarine (AFC) release from the fluorogenic substrate Ac-DEVD-AFC in the presence of cell lysates. The method was described in detail previously [23]. Following cell incubation with 20 μM IPA-3, the cells (3×10^6^) were washed, lysed and aliquots of cytosolic proteins were incubated with Ac-DEVD-AFC for 30 min at 37°C. Linear increase in fluorescence intensity at 520 nm was monitored using Fluostar Galaxy microplate reader (BMG Labtechnologies, Germany) and the slope was used as a relative value of caspase-3 activity.

### Measurement of Intracellular IPA-3 Content

The intracellular IPA-3 amount was measured as the increase in the mean fluorescence intensity upon 1 h cell incubation with IPA-3. The excitation wavelength was 405 nm, emission at 450 nm was detected (Pacific blue channel on BD LSRFortessa flow cytometer). Alternatively, the cells (6×10^6^) were collected by centrifugation, washed in PBS and the cell pellet was lysed in 50 μl lysis buffer (10 mM Hepes, pH 7.4, 2 mM EDTA, 0.1% CHAPS, 5 mM DTT) by repeated freezing and thawing (3 fold). The lysate was diluted by adding 450 μl DMSO and the fluorescence emission spectra (excitation at 405 nm) was recorded using Cary Eclipse fluorescence spectrophotometer (Varian). The spectrum of control cell sample was subtracted from the spectrum of IPA-3-treated sample.

### Measurement of Cell Adhesivity to Fibronectin

The end-point method for assessment of cellular adhesivity to fibronectin has been described previously [24]. Briefly, the cells (1×10^4^) were seeded into fibronectin-coated wells of a microtitration plate and incubated for 1 h at 37°C. Then, the wells were washed with PBS/Ca2+/Mg2+ using a multichannel adaptor to the suction-pump and the remaining cells were quantified by means of fluorescent labeling (Cy-Quant Cell Proliferation Assay Kit; Molecular Probes). The adherent cell fraction (ACF) was calculated using the fluorescence signal from fibronectin-coated plate and that from reference plate, which contained the total cell number.

### Real-time Microimpedance Measurement

The real-time analysis was performed as described previously [25], using RTCA XCelligence DP system from ACEA Biosciences (San Diego, CA, USA) which was placed in an incubator (at 37°C) with regulated CO_2_ content (5%). Fibronectin-coated E-plates containing 100 μl RPMI 1640 culture medium per well were equilibrated at 37°C and the electrical microimpedance signal (cell index) was set to zero in these conditions. The cells were added in 100 μl of suspension in RPMI 1640 in quadruplicates. IPA-3 stock solution was diluted in 50 mM Tris buffer and 4 μl were added to each well. DMSO diluted in Tris buffer was added to control wells.

### Interference Reflection Microscopy

Cells were incubated for 1 h on fibronectin-coated coverslip and fixed with 2% paraformaldehyde. The interference in reflected light was observed by means of FV-1000 confocal microscope (Olympus), using 405 nm laser beam and focusing to the coated glass surface.

### Immunoblotting

Polyacrylamide gel electrophoresis and western-blotting were performed as described previously [19] using antibodies against PAK kinases, stathmin or cofilin. Actin was used as the loading control.

### Cell Transfection

Jurkat cells (1 to 2×10^6^) were transfected with 20–28 pmol of siRNA targeting PAK1 or PAK2 or with 20 pmol of control (non-targeting) siRNA in 100 μl Nucleovette vessels, using program CL-120 in the 4D-Nucleofector System (Lonza, Basel). Cells were subsequently cultured for 24 h in RPMI 1640 (with or without antibiotics) prior to PAK expression level analysis and cell adhesivity measurement. The cell viability was assessed 24 and 48 h after nucleofection using propidium iodide exclusion assay. The measurement was performed using LSRFortessa flow cytometer (BD Biosciences).

## Results

### IPA-3 Reduces Cell Proliferation and Induces Cell Death in Human Hematopoietic Cells

**Figure 1:**
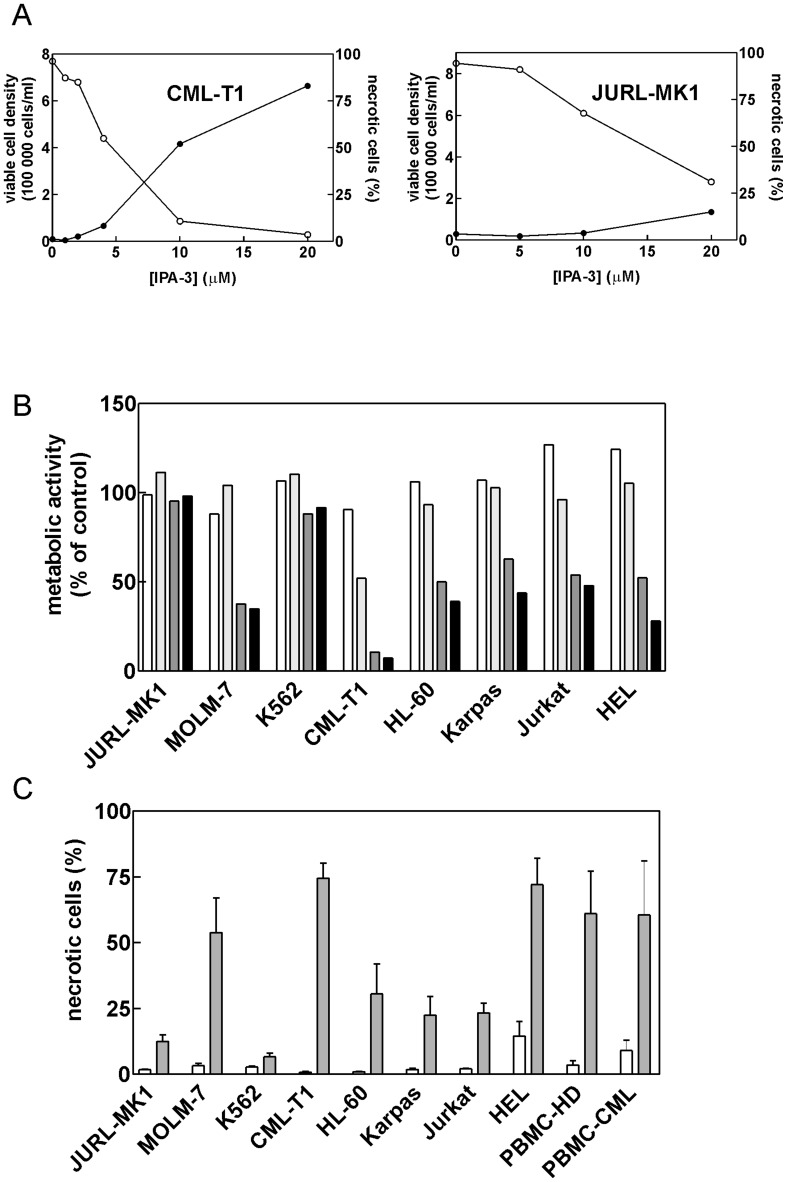
Effect of IPA-3 on cell growth and viability. (A) Cells were seeded at 2×10^5^ cells/ml and IPA-3 at different concentrations was added for 48 h. The number of viable cells (open symbols) and the necrotic cell fraction (closed symbols) were determined using Trypan blue exclusion test. CML-T1 and JURL-MK1 cells are shown as examples, the results for other cell lines are shown in Supporting Information, Figure S1. (B) Cells were seeded at 2×10^5^ cells/ml, incubated with 20 μM IPA-3 for 2 to 24 h and the metabolic activity was then measured using MTT assay as the percentage of the corresponding controls (untreated cells). White, light grey, dark grey and black bars correspond to 2, 10, 16 and 24 h incubation times, respectively. (C) Summary results from Trypan blue exclusion assay performed after 48 h incubation with 20 μM IPA-3. White bars: untreated controls, grey bars: IPA-3. At least 3 independent experiments were done for each cell line and for primary mononuclear cells from healthy donors (PBMC-HD), two experiments were performed using primary mononuclear cells from CML patients (PBMC-CML).

The effects of IPA-3 were studied in a panel of human cell lines derived from chronic myelogenous leukemia (JURL-MK1, MOLM-7, K562, CML-T1), acute myeloid leukemia (HL-60), acute lymphoblastic leukemia (JURKAT), erythroleukemia (HEL) and anaplastic large cell lymphoma (Karpas-299), as well as in human peripheral blood mononuclear cells (PBMC). IPA-3 treatment reduced the number of viable cells and induced cell death with EC50 ranging from 5 to more than 20 μM (Figure 1 A shows examples for CML-T1 and JURL-MK1 cells, similar results were obtained for other cell lines, see Figure S1). The cell metabolic rate in cells treated with IPA-3 was measured using tetrazolium reduction assay (MTT assay) for incubation times from 2 to 24 h. The most important decrease was observed between 10 h and 16 h time points for the majority of cell lines (Figure 1B). The fraction of unviable cells was determined using either Trypan-blue exclusion test (Figure 1C) or propidium iodide staining which gave closely similar results. CML-T1 cells were the most sensitive to IPA-3 effects whereas JURL-MK1 and K562 cells were rather resistant to up to 20 μM IPA-3 treatment (Figure 1, A–C). The cell death in immortalized cell lines was unusual in that Trypan blue-positive cells disintegrated very slowly and large round stained cells rather than cell debris were present in IPA-3-treated samples even after 72 h. Importantly, IPA-3 was also toxic for PBMC obtained either from healthy donors (N = 4) or from patients with chronic myelogenous leukemia (N = 2) (Figure 1C). However, the cell death of primary cells was associated with cell shrinkage and was followed by fast disintegration.

To account for possible contribution of oxidative stress generated by IPA-3, we tested the effect of a control compound, PIR3.5, which has similar structure as IPA-3 but does not inhibit PAK kinases. In the majority of cell lines, we observed only mild toxicity of 20 μM PIR3.5 after 48 h treatment (Figure S2). Quite surprisingly, for HL-60 cells, PIR3.5 was considerably more toxic than IPA-3 (about 90% necrotic cells after 48 h PIR3.5 treatment vs about 30% after IPA-3 treatment).

### IPA-3-induced Cell Death has Apoptotic Features

**Figure 2:**
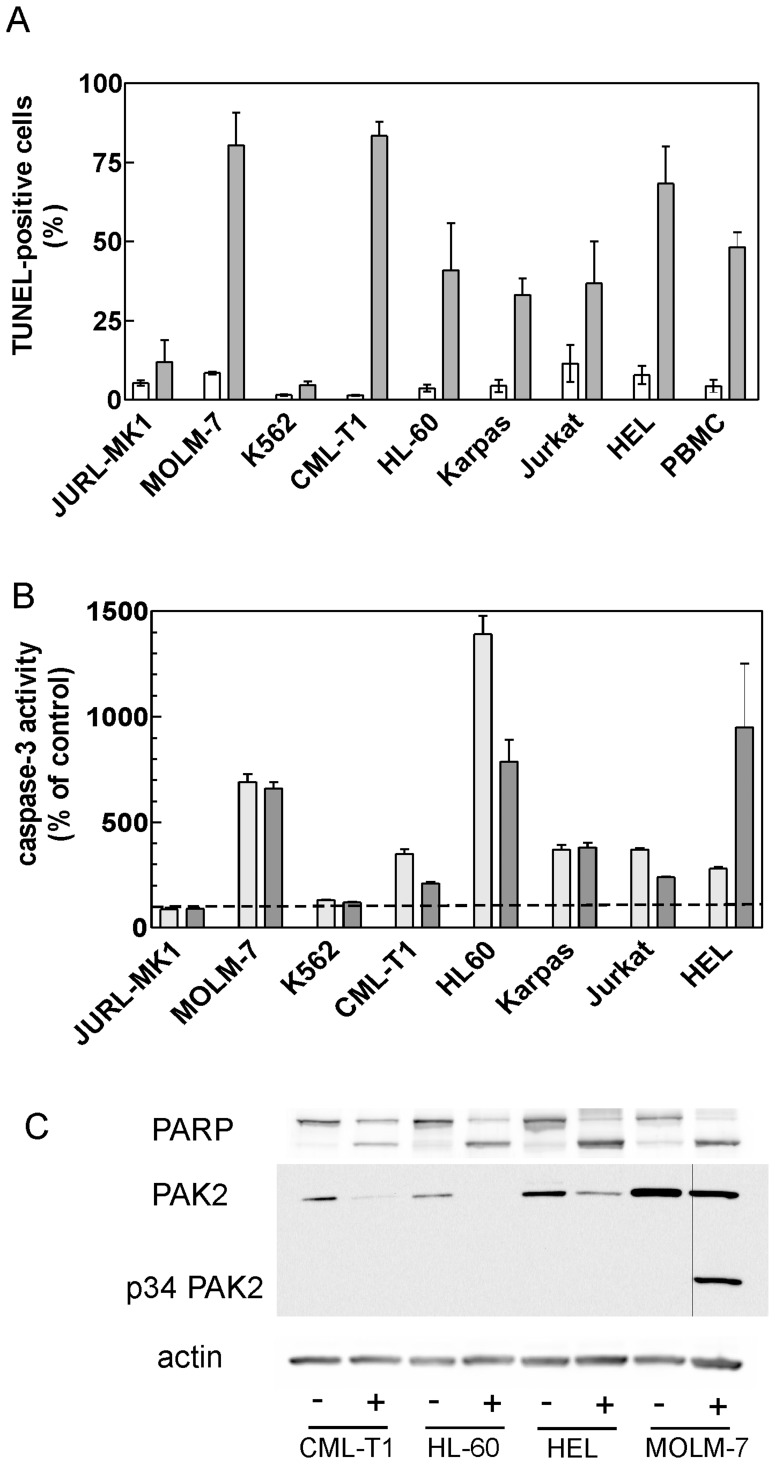
IPA-3-induced cell death displays apoptotic features. (A) Cells were treated for 48 h with 20 μM IPA-3 and apoptotic DNA breaks were detected using TUNEL assay. Clear bars: control cells, dark bars: IPA-3. The fraction of TUNEL-positive cells is shown as mean and range from 2–3 independent experiments. Primary mononuclear cells (PBMC) were isolated from the peripheral blood of a healthy donor. (B) Cells were treated for 13 h (light bars) or 23 h (dark bars) with 20 μM IPA-3, harvested and caspase-3 activity in cell lysates was assessed using the fluorogenic caspase-3 substrate Ac-DEVD-AFC. Caspase-3 activity in IPA-3-treated cells is shown as relative to the values obtained from the corresponding untreated controls. The dashed line highlights the control level (100%). (C) PARP and PAK cleavage was detected using western-blotting in control cells (−) and cells incubated with 20 μM IPA-3 (+). The incubation time was 13 h for CML-T1 and HL-60 cells and 24 h for HEL and MOLM-7 cells.

IPA-3-induced cell death was accompanied by generation of DNA strand breaks that are characteristic of the apoptosis and are detected using TUNEL assay (Figure 2A). Again, only low fraction of TUNEL-positive cells was detected in JURL-MK1 and K562 cell lines. Caspase-3 was activated by IPA-3 treatment in the majority of cell lines (Figure 2B). However, the caspase inhibitor Q-VD-OPh had only limited effect on IPA-3-induced processes: both apoptotic DNA cleavage and cell death were reduced by 10 to 30% in the majority of cell lines (by 45% in MOLM-7) when the cells were treated with 20 μM Q-VD-OPh in addition to IPA-3 (Figure S3). Selected cell lines with marked caspase-3 activation were also tested for cleavage of the known caspase-3 substrate, PARP, and for possible formation of p34 fragment of PAK2 which was reported to be generated during the apoptosis (Figure 2C). Indeed, PARP cleavage was detected in all the tested IPA-3-treated cells. The formation of p34 PAK fragment was evident in MOLM-7 cells (Figure 2C). Weak band at the same position was detectable using long integration times in IPA-3-treated HL-60 and HEL cells.

### IPA-3 does not Induce G2/M Cell Cycle Arrest

The kinase PAK1 was reported to be involved in the regulation of cell cycle progression through the mitotic checkpoint [26]. We analyzed the DNA content distribution in cells treated for 24 h or 48 h with 20 μM IPA-3 using propidium iodide staining and did not found any increase in the cell fraction in G2/M phase due to IPA-3 treatment, in any of the cell line studied (Figure S4). The most marked effect was the transfer of apoptotic cells into sub-G1 region. Otherwise, an increase in the relative cell amount in G1 phase was usually observed but the difference was statistically significant only for JURL-MK1 and HL-60 cells.

### The Intracellular IPA-3 Content Differs among Different Cell Types

**Figure 3:**
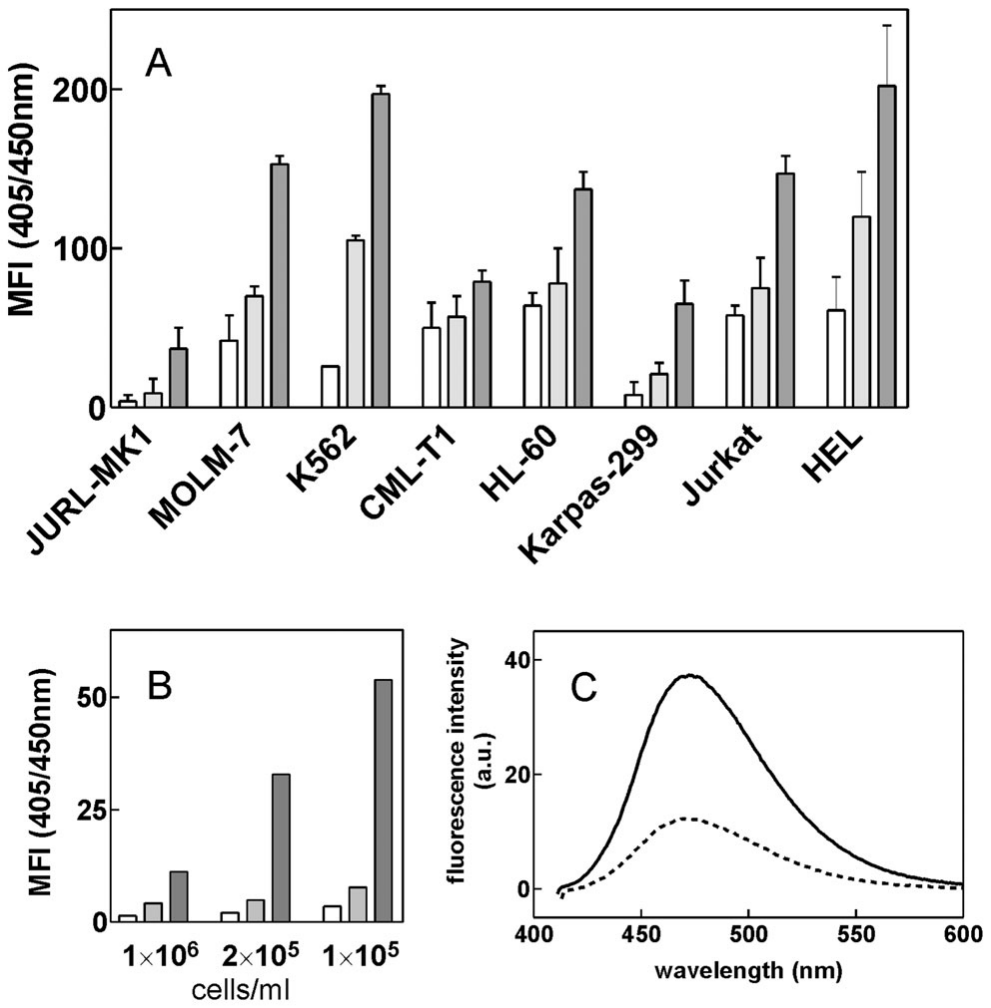
Intracellular IPA-3 amount determined using flow cytometry or fluorescence spectroscopy. (A, B) Results from flow cytometry measurements. The relative amount of IPA-3 was obtained as the increase in the mean fluorescence intensity (MFI) at 405/450 nm (excitation/emission) from samples incubated for 1 h with IPA-3 at 5, 10 or 20 μM extracellular concentration (white, light grey and dark grey bars, respectively) compared to the untreated control samples. (A) Cells were seeded at 2×10^5^ cells/ml, the figure shows means and standard deviations from 3 independent experiments. (B) JURL-MK1 cells were seeded at different cell density as indicated and incubated for 1 h with IPA-3. (C) MOLM-7 cells were seeded at 6×10^5^ (dotted line) or 1×10^5^ cells/ml (solid line) and incubated for 1 h with 20 μM IPA-3 (extracellular concentration). Cell aliquots (6×10^5^ cells from each sample) were lyzed and suspended in DMSO and fluorescence spectra were recorded with the excitation wavelength set to 405 nm. The background fluorescence (sample from the same amount of untreated cells) was subtracted. Closely similar results were obtained in repeated experiments.

The intracellular amount of IPA-3 in different cell types was assessed through the fluorescence signal emitted by IPA-3 in the ultraviolet light range which was measured using a flow cytometer. The fluorescence was excited at 405 nm where only the functional (non-reduced) form of IPA-3 should be detected [16]. Figure 3A shows the increase in mean fluorescence intensity at 450 nm after 1 h incubation of cells with increasing IPA-3 concentrations. Considerable differences in IPA-3 content among different cell types were detected. Especially, JURL-MK1 accumulated significantly less IPA-3 than JURKAT, HL-60 or MOLM-7. Closely similar pattern was obtained when the excitation wavelength was set to 355 nm (in this case, both the reduced and the oxidated forms are excited). The kinetics of increase in UV fluorescence signal after IPA-3 addition was fast (the halftime was of about 4 min), the equilibrium state being reached in about 20 min (data not shown). The intracellular amount of IPA-3 also varied as a function of the cell density for some cell lines. Figure 3B shows the relative IPA-3 amount in JURL-MK1 cells seeded at different cell density and incubated for 1 h with IPA-3 at the same extracellular concentration. Similar effect (higher intracellular IPA-3 content resulting from less dense cell suspension) was observed when IPA-3 amount was determined spectrofluorimetrically from MOLM-7 cell lysates (Figure 3C).

### IPA-3 Treatment Affects Cellular Adhesivity to Fibronectin

**Figure 4:**
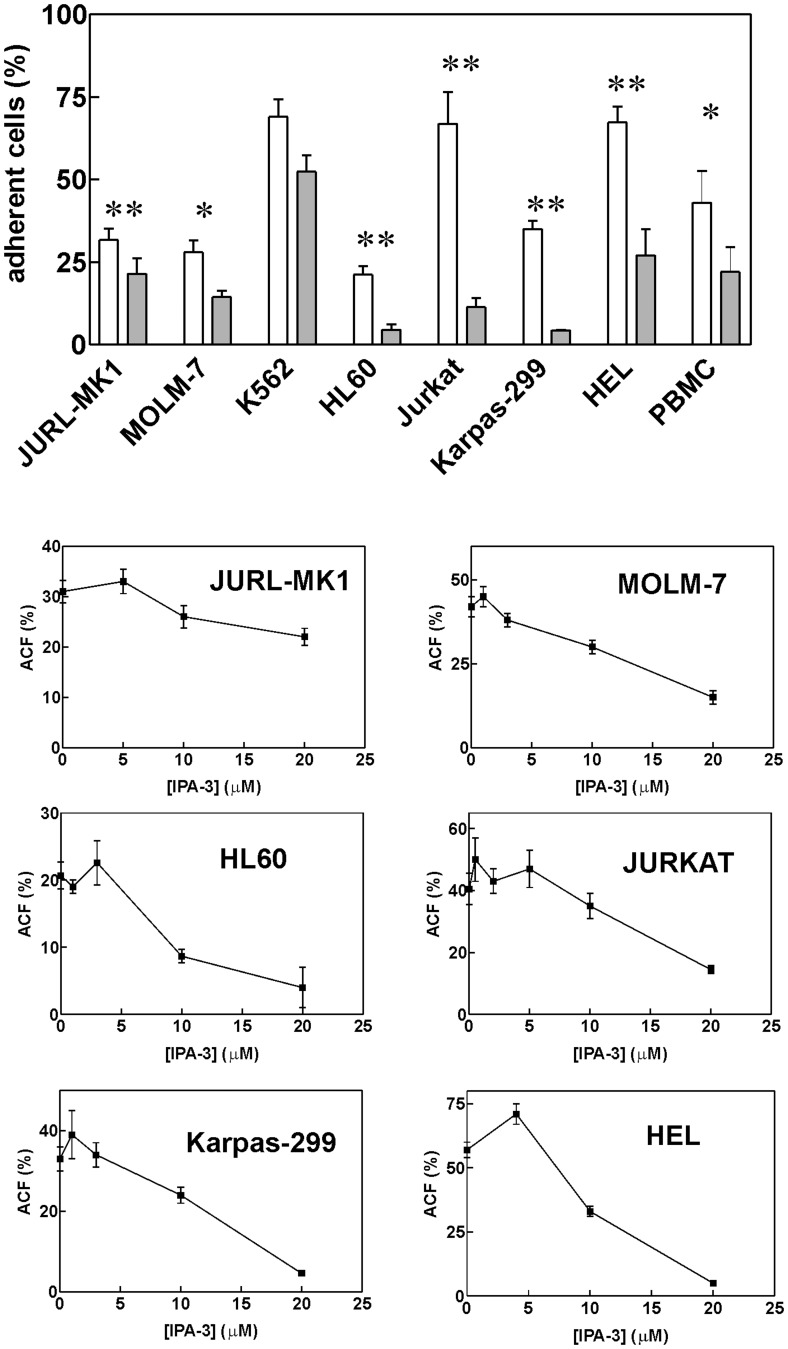
IPA-3-induced decrease in cell adhesivity to fibronectin. Top: Cells were treated for 2 h with 20 μM IPA-3 prior to 1 h incubation on fibronectin-coated surface. The fraction of adhered cells is shown as mean and standard deviation from independent experiments for each cell line (N = 3 to 5) as well as for peripheral blood mononuclear cells (PBMC) obtained from healthy donors (N = 3) or from leukapheresis (N = 2). White bars: untreated controls, grey bars: 20 μM IPA-3. The differences between treated samples and controls were statistically significant (*P<0.05, **P<0.01) with the exception of K562 cells. Lower graphs: Dose dependency of IPA-3-induced loss of cell adhesivity to fibronectin. ACF - adherent cell fraction.

We have noted previously that 20 μM IPA-3 pretreatment reduced subsequent JURL-MK1 cell binding to fibronectin-coated surfaces [19]. Similar results were obtained for the majority of cell types studied in this work, including primary cells (Figure 4). CML-T1 cells are very poorly adherent even without treatment [25] and no significant effect of IPA-3 could thus be discerned. Beside this, the difference between controls and cells treated with 20 μM IPA-3 was statistically significant, except for K562 cells (Figure 4A, p = 0.07, N = 5 for K562). Dose-response curves for IPA-3-induced drop in the cell adhesivity to fibronectin (FN) are shown in Figure 4 (lower part). At low IPA-3 concentrations, a small increase in the cell adhesivity was usually observed.

Figure 4 shows the results obtained for 2 h treatment time, but we also found that 10 min treatment with 20 μM IPA-3 was sufficient to obtain 4fold reduction of the subsequent binding of Jurkat cells to FN. On the other hand, the effect of IPA-3 was much less pronounced when the cells were allowed to adhere to FN-coated surface prior to IPA-3 addition. In this case, IPA-3 had no effect on the adhered cell fraction in MOLM and HEL cells and several hours were required to obtain a decrease in the adhered cell fraction in Karpas, HL-60 or Jurkat cells (e.g. reduction to 65% of the control value after 150 min incubation of Jurkat cells with 20 μM IPA-3, not shown).

While the majority of cells used in this study (JURL-MK1, MOLM-7, K562, HL-60) exclusively adhere to FN, some of them also bind to other ECM proteins (Table S1). Especially, HEL cells adhere to laminin and collagens in addition to FN. The adhesion to these ECM proteins was also found to be strongly reduced by 20 μM IPA-3 treatment (Figure S5).

**Figure 5:**
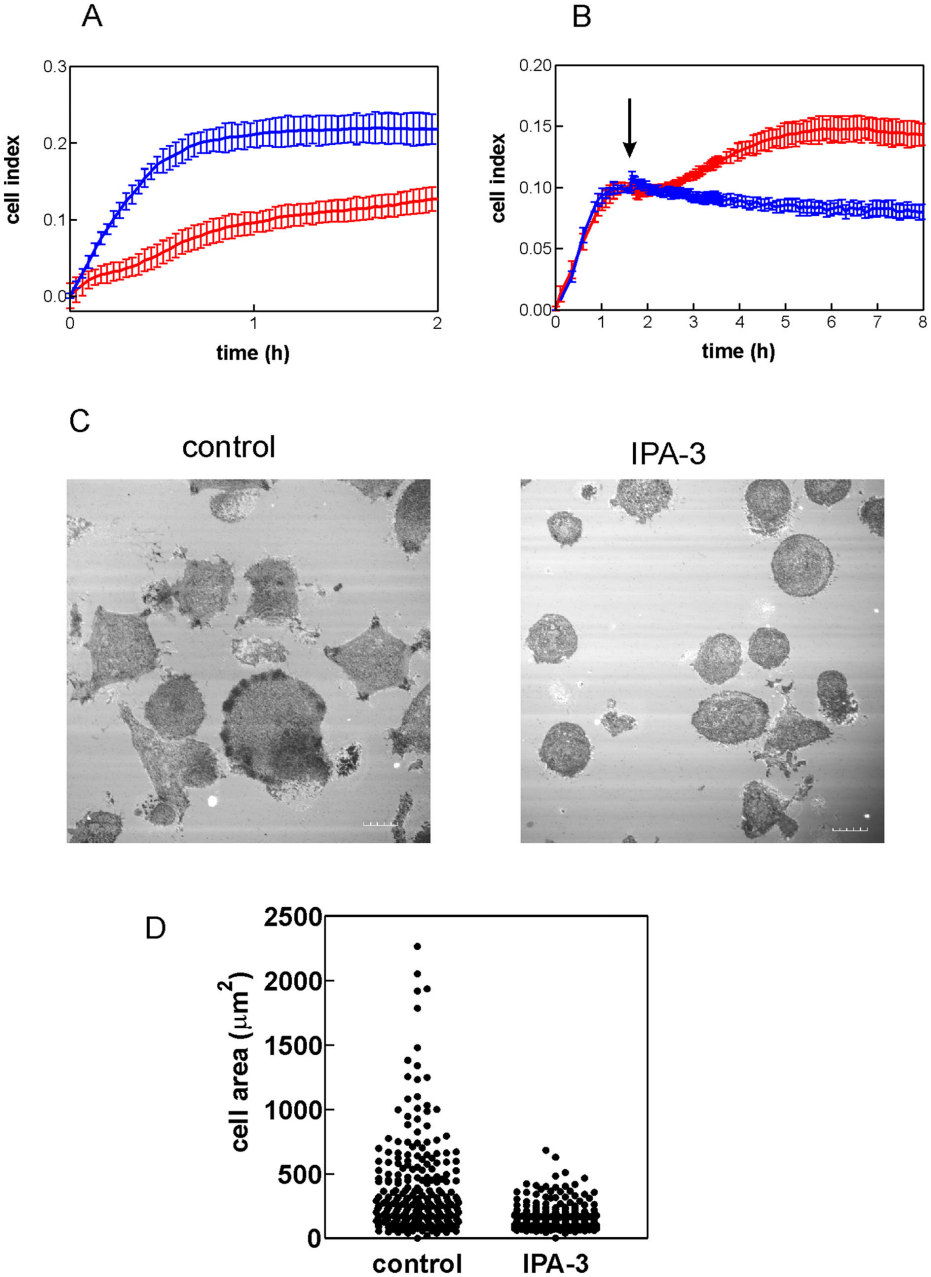
Effect of IPA-3 on HEL cell interaction with fibronectin-coated surface. (A) HEL cells (at 1×10^5^ cells/ml) were pretreated for 30 min with 20 μM IPA-3 and the same amount (60 000) of control (blue) and IPA-3-treated (red) cells was applied to FN-coated microplate with embedded microelectrodes. The observed changes in microimpedance reflect cell binding to the cell bottom. (B) HEL cells (20 000/well) were applied to FN-coated microplate and allowed to adhere for about 90 min. Then IPA-3 was added at the time point indicated by the arrow. Blue: DMSO only, red: IPA-3 (20 μM final concentration in the well). Curves in A and B show means and standard deviations of wells in quadruplicates. (C) Analysis of HEL cells interacting with FN-coated coverslips by interference reflection microscopy. The cells were pretreated with 20 μM IPA-3 (right panel) or left untreated (left panel) prior to incubation for 1 h on FN-coated coverslip (at 37°C). Scale bars: 10 μm. (D) Comparison of cell areas for control and IPA-3-treated HEL cells. The experiment was performed as in (C) and the cell area was measured for about 250 cells (all cells in multiple randomly chosen fields).

As we have reported recently, the kinetics of cell interaction with FN-coated surface can be monitored using real-time microimpedance measurement [25]. The assay is based on measurement of electrical impedance between two interdigitated systems of microelectrodes which are embedded on the bottom of the wells of a microtitration plate. Cell adhesion to the well bottom results in an increase of the measured microimpedance signal. Cells growing in suspension do not generate any appreciable signal change unless the cell bottom is coated with FN or other cell-binding protein. Figure 5A shows the increase of microimpedance following addition of HEL cells to FN-coated wells. In agreement with the above mentioned findings (Figure 4), HEL cells pretreated for 30 min with 20 μM IPA-3 attached to FN markedly more slowly in comparison with untreated cells. On the other hand, IPA-3 had rather opposite effect if added only after the cell attachment to FN (Figure 5B). In this case, no decrease in microimpedance was observed up to 8 h following IPA-3 treatment of HEL cells. Similar behaviour has been observed in MOLM-7 cells.

The effect of IPA-3 was also evident during cell imaging in interference reflection microscopy (IRM). This method allows for visualization of cell parts that are in close contact with the glass surface [27]. No IRM signal is obtained from leukemic cells on bovine serum albumin (BSA)-coated coverslips while cell binding to FN generates dark areas corresponding to the zones of attachment. As it is shown in Figure 5C and D, IPA-3 pretreatment (30 min, 20 μM) largely reduced cell spreading on FN-coated glass coverslips.

We wondered if the cell binding to FN can protect the cells against IPA-3 toxicity. Using the MTT assay, we compared the effect of IPA-3 in the presence and in the absence of FN. The cells were seeded into wells coated with FN (or BSA as a control), incubated for 1 h at 37°C and then treated with 20 μM IPA-3 for 20 h prior to MTT addition. IPA-3 reduced the metabolic activity to the same extent regardless of the coating (data not shown).

### Group I PAK Expression Levels in Leukemic Cell Lines

**Figure 6:**
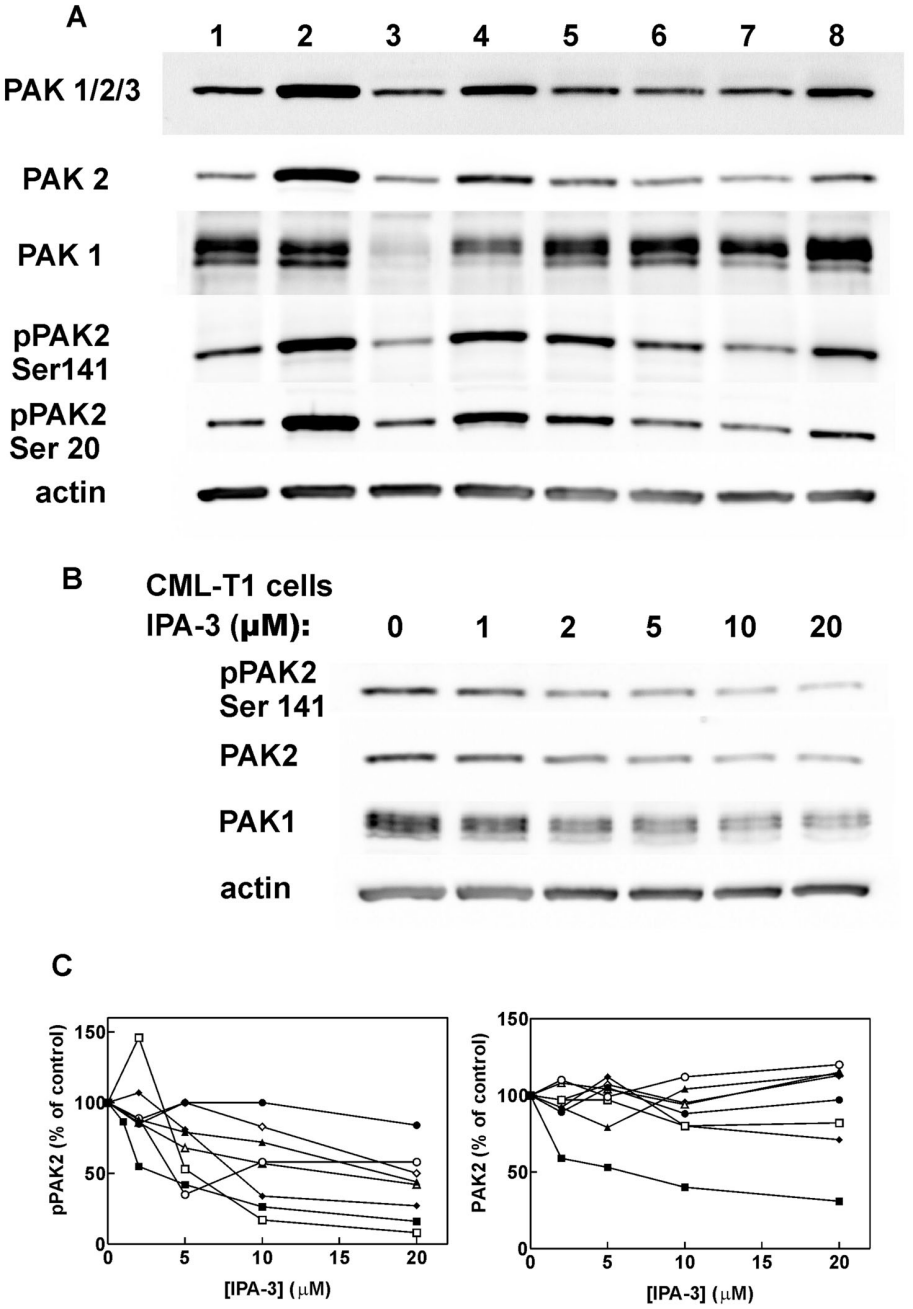
Expression of PAK kinases in the studied cell lines. Cell proteins were separated using gel electrophoresis and the expression levels of PAK1, PAK2, phospho-PAK (Ser 144/Ser141) and phospho-PAK2 (Ser20) were determined using western-blotting. (A) Lanes 1–8 correspond to the individual cell lines: (1) JURL-MK1, (2) MOLM-7, (3) K562, (4) CML-T1, (5) HL60, (6) Karpas-299, (7) Jurkat, (8) HEL. (B) CML-T1 cells were treated with IPA-3 at the indicated concetrations for 2 h, then lysed and subjected to electrophoresis and western-blotting. (C) Relative expression levels of phospho-PAK2 (Ser141) and PAK2 in cell lines treated for 2 h with IPA-3 at different concentrations. The band intensity from western-blots with anti-PAK antibodies was corrected for small differencies in protein loads using actin bands and the resulting values were expressed as relative to controls (without treatment). JURL-MK1:closed circles, MOLM-7:closed triangles, CML-T1:closed squares, K562:open circles, HL60:open squares, JURKAT:open triangles, Karpas-299:open diamonds, HEL:closed diamonds.

The expression level of group I PAKs was studied using a set of antibodies against these kinases. The antibody recognizing all group I kinases (PAK1, 2 and 3) detected one dominant band at 60 kDa (Figure 6A). The same band was also detected using the specific anti-PAK2 antibody (Figure S6). Although both anti-PAK1/2/3 and anti-pPAK (Ser144/Ser141) antibodies recognize PAK1 (datasheet from the provider), no band at PAK1 position was detected in lysates from leukemic cell lines using these two antibodies. Thus, PAK1 expression was very low compared to that of PAK2. Nevertheless, PAK1 expression was detected at higher molecular weight (about 68 kDa) using the specific anti-PAK1 antibody. Interestingly, PAK1 level was markedly lower in K562 cells (lane 3 in Figure 6A) compared to the other cell lines. No specific signal was obtained from anti-PAK3 antibody (e.g. Figure S6 for MOLM-7 cells). The phosphorylation status of PAK2 Ser141 and/or Ser20 (autophosphorylation sites) reflects PAK kinase activity. The highest level of p-PAK2(Ser141) was found in MOLM-7 cells (lane 2) while in K562 cells, both PAK2 and p-PAK2 levels were low. The levels of p-PAK2 and of total PAK2 in samples of CML-T1 cells treated for 2 h with different doses of IPA-3 are shown in Figure 6B. In CML-T1 cells, both p-PAK2 and PAK2 levels, as well as that of PAK1, were reduced by the treatment. In the majority of cell lines other than CML-T1, we observed a decrease for Ser141 phosphorylation status but not for the total PAK2 level after 2 h treatment with IPA-3 (Figure 6C and Figure S7). Figure 6C shows that Ser141 dephosphorylation is progressive in the concentration range from 2 to 20 μM IPA-3. Note that a decrease in total PAK level usually occurred later, probably in relation to the proceeding apoptosis (Figure 2C).

### IPA-3 Reduces Cofilin Phosphorylation at Ser3

**Figure 7:**
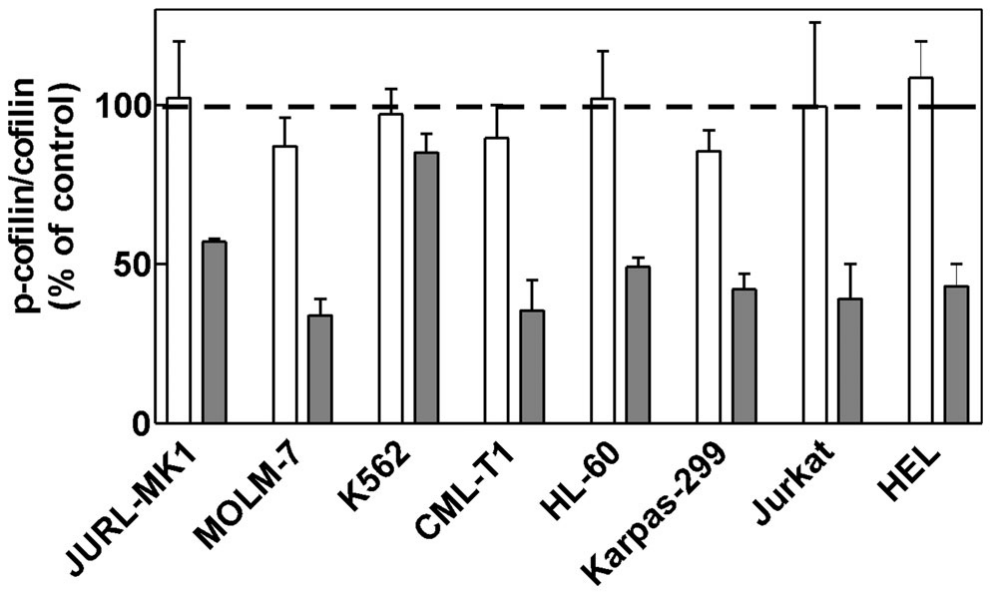
Effect of IPA-3 on cofilin phosphorylation at Ser3. Cells were incubated for 2 μM IPA-3 (white bars and dark bars, respectively), lysed and the level of cofilin and phospho-cofilin (Ser3) was determined by western-blotting. The level of total cofilin remained essentially unchanged and was used as the loading control. Phospho-cofilin/cofilin ratio was expressed as relative to the value obtained from untreated controls (100%). The graph shows means and standard deviations from repeated independent experiments.

To assess the dose dependency of IPA-3 impact on PAK activity we also explored the phosphorylation status of a known downstream PAK effector, cofilin. As it is shown in Figure 7, 2 h treatment with IPA-3 reduced cofilin phosphorylation at Ser3 with EC50 situated usually between 5 and 20 μM, except for JURL-MK1 and K562 cells. We have also tested the phosphorylation status of a known PAK target, stathmin, at Ser16, but we failed to detect any change in p-stathmin level following IPA-3 treatment (tested in all cell lines included in this study, data not shown).

### PAK Silencing using siRNA

**Figure 8:**
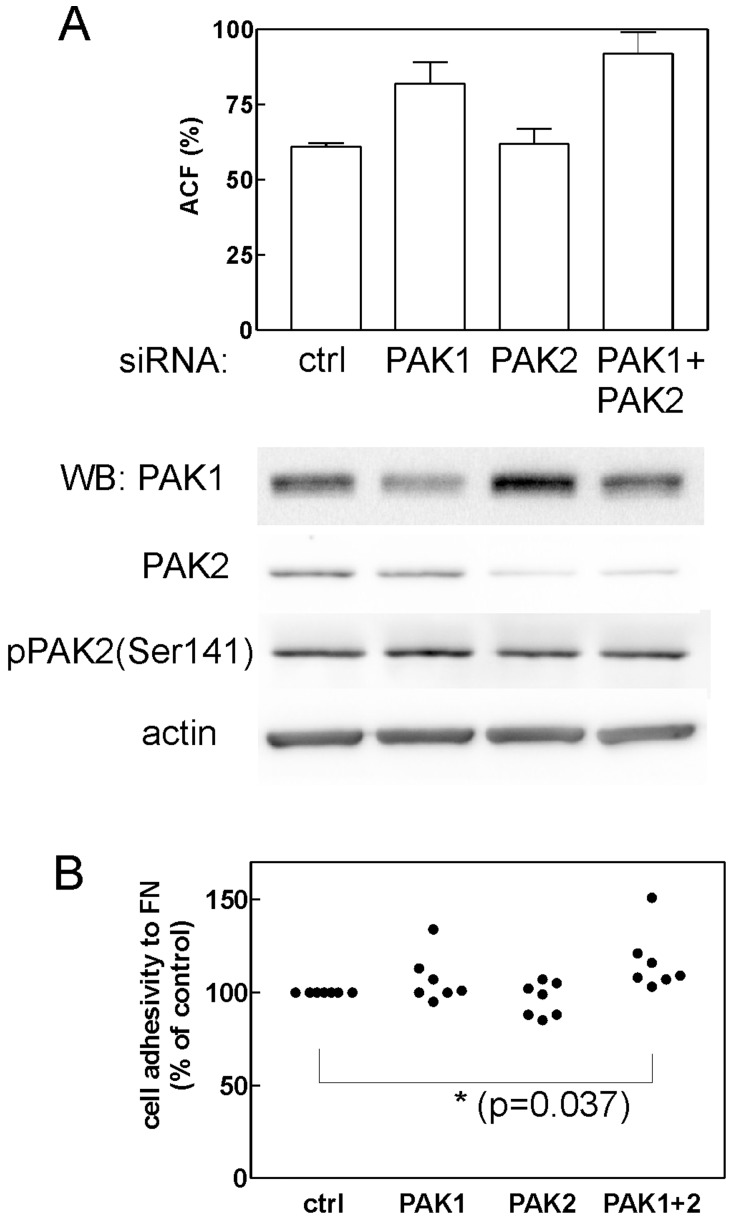
Effect of anti-PAK siRNAs on JURKAT cell adhesion. Cells were nucleofected with 2 μl esiRNA against PAK1 or PAK2, with the combination of both esiRNAs (PAK1+PAK2) or with non-targeting siRNA (ctrl) and the cell adhesion to fibronectin (ACF- adherent cell fraction) was tested 22 h post nucleofection. The level of PAK1, PAK2 and pPAK2 (Ser141) was assessed at the same time by western-bloting. (A) The experiment with the highest silencing efficacy obtained. The graph of cell adhesivity shows means and standard deviations of sample quadruplicates. (B) Summary of results for all adhesion experiments (N = 7). The adherent cell fraction was expressed as relative to the adhesivity of controls and the resulting values were compared using paired Student’s t-test.

To further specify the role of PAK1 and PAK2 in cell adhesivity and cell death, we attempted to silence these kinases in Jurkat cells using small interfering RNAs (siRNAs). Two different siRNA sources were tested (mission esiRNAs from Sigma were found to be more efficient than siRNAs from Cell Signaling), but only partial reduction of PAK expression was achieved (Figure 8A). The maximal reduction of PAK1 level was by 40% at 24 h after the nucleofection (the effect was lower at later time points). A larger decrease was apparently obtained for PAK2 (reduction by 78%). However, the amount of the active form of the kinase (phosphorylated at Ser141) was lowered only by 20%. Accordingly, no significant difference in cell viability was observed after anti-PAK1, anti-PAK2 or combined anti-PAK1+anti-PAK2 siRNA nucleofection compared to control siRNA nucleofection. On the other hand, the decrease of PAK level was associated with an increase in the cell adhesivity to fibronectin. This effect was statistically significant for the combined PAK1+PAK2 inhibition (Figure 8B).

## Discussion

The effect of group I PAK inhibition using IPA-3 [26], [28], dominant-negative interacting domain [8] or siRNA [3], [8], [11], [28]–[32] has been described for various adherent cell types. While no cytotoxicity was reported following individual PAK targeting by means of dominant negatives or siRNA, toxic effects of IPA-3 treatment were noted in melanoma and colon carcinoma cell lines [28]. Gene knockout experiments support the view that PAK1 is not required for the cell viability as PAK1-knocked-out mice are healthy and fertile although they may suffer from defects in the immune system function [1], [2]. On the other hand, PAK2 knockout leads to early embryonic lethality in mice [20], [33]. PAK2 knockdown using shRNA also slowed down the proliferation of skin cancer cell lines [34]. We found that the small molecule inhibitor IPA-3 induces cell death in the majority of the studied human leukemic cell lines as well as in primary blood cells (Figure 1). The cell death had apoptotic features as it involved caspase-3 activation, PARP cleavage and formation of apoptotic DNA breaks (Figure 2). However, we also noted that the cell integrity was often preserved in IPA-3-treated cells despite the loss of the cell viability. This might be due to inhibition of pro-apoptotic action of PAK2. It is known that the activated form of PAK2 (p34 fragment) resulting from PAK2 cleavage by caspases is a proapoptotic effector that mediates morphological changes seen in apoptosis [30], [33], [35], [36]. We actually detected formation of p34 PAK2 fragment in MOLM-7 cells (Figure 2C). However, it is not known if the activity of this fragment can be inhibited by IPA-3 as p34 lacks the N-terminal regulatory domains that are implicated in the autoregulation.

PAK1 was reported to be required for cell entry into mitosis [26] and we thus searched for possible cell cycle arrest in G2/M phase following IPA-3 treatment. However, the cell cycle analysis rather indicated an increase in G1/G0 phase or no change in the cell cycle phase distribution (Figure S4). This suggest that IPA-3 can induce cell death without cell cycle arrest in the mitotic checkpoint.

The caspase inhibitor Q-VD-OPh has high potency in inhibiting the proteases of caspase family. We have previously shown that 10 μM Q-VD-OPh fully inhibits caspase-3 and -7 activity, formation of apoptotic DNA breaks and decrease in cellular adhesivity to fibronectin in JURL-MK1 and HL-60 cells treated with apoptosis-inducing drugs [37]. On the other hand, we noted in the present work that Q-VD-OPh had usually only minor effect on the processes induced by IPA-3 (Figure S3). It is thus possible that multiple cell death pathways including caspase-independent apoptosis are triggered in parallel by IPA-3. In MOLM-7, the relative contribution of caspase-dependent apoptosis to the cell death is larger than in other cell lines (Fig. S3). This may be the reason for more marked formation/accumulation of p34 PAK fragment which is specifically due to caspase activity.

A recent work [20] has shown that group I PAKs are required for the engraftment of hematopoietic stem cells in the mouse model. Inhibition of group I PAK expression by dominant-negative PAK-interacting domain (PID) completely abrogated the ability of hematopoietic stem cells to restore the hematopoiesis in irradiated animals. In gene knock-out experiments, PAK2, but not PAK1, was found to be required for reconstitution of hematopoiesis in irradiated murine recipients. Our analysis of the effects of PAK inhibition with IPA-3 suggests that the reduced engraftment may be due to the loss of the cell ability to bind to the bone marrow extracellular matrix (Figures 4, 5 and S5). The changes in the cell adhesivity to fibronectin occured very early following IPA-3 addition. This indicates that it is a direct consequence of kinase inhibition not related to the apoptosis. Interestingly, IPA-3 was less efficient when it was added to cells interacting with fibronectin (Figure 5B). This may be associated with the fact that the compound binds only to the inactive PAK molecules and prevents their activation while it is not able to inhibit the active PAK pool.

The compound PIR3.5 is proposed to be a control for possible contribution of oxidative stress to the effects of IPA-3. We found that PIR3.5 was indeed slightly toxic for the majority of leukemic cells while it had hardly any effect on the cell adhesion to fibronectin (Figure S2). Oxidative stress generation is thus not the major mechanism of action for the observed IPA-3-induced effects. In addition, HL-60 cells were markedly more sensitive to PIR3.5 than to IPA-3 which indicates that PIR3.5 may not be suitable control compound for IPA-3.

Although IPA-3 is considered to be specific in PAK inhibition [16], we cannot rule out the possibility that the induction of cell death and the loss of cellular adhesivity in hematopoietic cells are due to other IPA-3 targets. Cells growing in suspension are known to be hard to transfect and, unfortunately, we were not able to achieve a substantial reduction of PAK1/PAK2 level using siRNA. The partial reduction of PAK levels resulted in an increase in the cell adhesivity to fibronectin (Figure 8), in agreement with the effect of lower IPA-3 doses. The effects observed at 20 μM IPA-3 may be due to complete inhibition of PAK1, PAK2 or both. Alternatively, IPA-3 may have yet unidentified additional target which is required for cell binding to extracellular matrix proteins. Nevertheless, inhibition of PAK activity (Figure 6B and C) and reduction of cofilin phosphorylation at Ser3 (Figure 7) have similar EC50 values as IPA-3-induced cell death (Figures 1A and S1) and loss of adhesivity to fibronectin (Figure 4). In Figure 6C, PAK dephosphorylation appears to be incomplete even at 20 μM IPA-3 for the majority of cell lines. However, this may be due to unspecific background staining as phospho-specific antibodies usually recognize multiple phosphorylated proteins. This background is cell type-specific and may distort the real extent of protein inhibition. On the other hand, a pool of PAK molecules may exist which would not be inhibited after 2 h incubation with IPA-3, either due to permanent activation (IPA-3 does not bind to the active form of the kinase) or inaccessibility (complex formation with other cellular components). Nevertheless, Figures 6C and 7 give evidence of progressive decrease of PAK activity in the concentration range from 2 to 20 μM.

A possible cause of difference in IPA-3 efficiency among various cell types is unequal pharmacokinetics. We analyzed the intracellular IPA-3 content after 2 h incubation with IPA-3 and found significant differences that can only partially be explained by different cell size (Figure 3). As indicated by the main cell diameter (see Material and Methods), the majority of cells included in this study have approximately the same size, while the volume of CML-T1 cells is about 3fold smaller and that of K562 and HEL cells about 2fold larger compared to JURL-MK1. Thus, the intracellular IPA-3 concentration in JURL-MK1 cells is relatively low compared to the other cell lines and, on the other hand, CML-T1 accumulate higher IPA-3 amount. The results presented in Figure 3 also suggest that an active mechanism must be involved in IPA-3 uptake or exclusion from the cells. Theoretically, the observed differences in IPA-3 intracellular content could also be due to different rate of disulfide bond reduction in the intracellular environment. However, similar differences among cell lines were found when the excitation was set to 355 nm where both intact IPA-3 and the reduced product are excited and emit fluorescence signal.

The results obtained for the eight leukemic cell lines are summarized in Table 1. It appears that IPA-3 toxicity depends from PAK expression levels as well as from the extent of drug accumulation. K562 cells, which are relatively resistant to IPA-3-induced cell death, express low levels of PAK1 and PAK2 (Figure 6A). Cell lines with high PAK levels (HEL and MOLM-7) are the most sensitive to IPA-3 treatment. Also, CML-T1 cells achieve the highest IPA-3 intracellular concentration (given their small size) and are very sensitive to IPA-3-induced cell death. On the other hand, the relative resistance of JURL-MK1 cells is probably due to low IPA-3 uptake in this cell line.

**Table 1:** Summary of results obtained for the individual leukemic cell lines treated with 20 μM IPA-3.

Cell line	PAK1 expression	PAK2 expression	intracellular IPA-3	effect on cell viability	effect on cell adhesivity to FN
JURL-MK1	++	+	+	+	+
MOLM-7	++	+++	++	+++	++
K562	+/−	+	++	+/−	+
CML-T1	+	++	+++	+++	not adherent
HL60	++	++	++	++	+++
Karpas-299	++	+	++	++	+++
JURKAT	++	+	++	++	+++
HEL	+++	++	++	+++	++

The protein expression, intracellular IPA-3 content and cell response to treatment were classified as low (+), medium (++) or high (+++) with regard to the range of the observed values from the whole set of cell lines. To assess IPA-3 intracellular concentration, the measured values for mean fluorescence intensity (shown in Fig. 3A) were corrected for differences in the cell volume estimated from the measured cell diameter.

The regulatory mechanisms of hematopoietic cell adhesion to the extracellular matrix are not well described and both similarities and differences with regard to adherent cell types were reported [38]–[40]. Kinases PAK (effectors of Rac1 and Cdc42 family of small GTPases) and ROCK (effectors of RhoA) are important regulators of cytoskeleton dynamics in adherent cells. PAK1 was also shown to act as downstream effector of RhoH, which is specifically expressed in hematopoietic cells and regulates T-cell migration [41]. Both PAK and ROCK activities inhibit F-actin severing and degradation through LIM kinase-mediated inactivating phosphorylation of cofilin at Ser3. In adherent cells, inhibition of ROCK activity leads to F-actin disassembly and loss of cellular adhesivity to the extracellular matrix. We have previously shown that in leukemic cell lines, the effect of ROCK inhibition by Y-27632 on cellular adhesivity to fibronectin was cell type-dependent and rather mild even when the compound significantly lowered cofilin phosphorylation [24]. Y-27632 did not affect cell proliferation and no toxicity was noted in any of the cell lines studied. On the other hand, the results presented in this work suggest that PAKs play an important role in the formation of cell-matrix adhesion structures and in the regulation of cofilin activity in hematopoietic cells (Figure 7). One can note that in K562 cells, the absence of IPA-3 effect on cofilin Ser-3 phosphorylation is in line with low PAK expression level.

Group I PAKs were reported to phosphorylate the microtubule-regulating protein, stathmin, in adherent cells [42], [43] as well as in mast cells [44]. However, we found that stathmin phosphorylation at Ser16 is not affected by PAK inhibition in leukemic cell lines.

In conclusion, our study shows that IPA-3 is toxic for the majority of leukemic cell lines as well as for primary hematopoietic cells. In addition, it strongly reduces cell binding to extracellular matrix proteins. Although the observed effects could be associated with inhibition of unspecific targets, indirect proofs relate IPA-3 toxicity to PAK inhibition. Indeed, cells with higher PAK expression are more sensitive to IPA-3-induced cell death and the observed effects on the cell behaviour occur in the same concentration range as PAK inhibition and cofilin dephosphorylation. Our results indicate that PAK kinases are probably required for the formation of dynamic adhesion structures during hematopoietic cell binding to the extracellular matrix.

## Supporting Information

Figure S1 **Dose-response curves for IPA-3 effects on cell growth and viability of different cell lines.** Cells were seeded at 2×10^5^ cells/ml and treated for 48 h with IPA-3 at different concentration as indicated. Viable cell density (open symbols) and fraction of necrotic cells (closed symbols) were determined by cell counting using Trypan blue exclusion test.
(TIF)

Figure S2 **Effect of PIR3.5 on the viability and adhesivity of leukemic cell lines.** Cells were treated with 20 μM PIR3.5 which is used as a control for IPA-3. White bars: controls (cells treated with DMSO), dark bars: cells treated with PIR3.5. A: effect of 48 h PIR3.5 treatment on the cell viability, means and s.d. from 3 independent experiments. B: effect of 2 h PIR3.5 treatment on the cell adhesivity to fibronectin, means and s.d. from sample quadruplicates.
(TIF)

Figure S3 **Effect of caspase inhibitor Q-VD-OPh on IPA-3-induced apoptotic DNA fragmentation.** Cells were treated for 48 h with 20 μM IPA-3 alone (clear bars) or in combination with 20 μM Q-VD-OPh (dark bars) and apoptotic DNA breaks were detected using TUNEL assay.
(TIF)

Figure S4 **Cell cycle analysis of control and IPA-3-treated cell lines.** Cells were treated with 20 μM IPA-3 for 24 h, the cell fraction in sub-G1 phase is shown on the left. The fraction of cells in G1/G0, S and G2/M phase are expressed as relative to G1/G0+S+G2/M (cells not in sub-G1) and shown on the right. CML-T1 cell line is mixed diploid/tetraploid and the cell cycle distribution thus cannot be derived from DNA content profiles. Means and s.d. from 3 independent experiments. White bars: controls, dark bars: IPA-3.
(TIF)

Figure S5 IPA-3 reduces HEL cell adhesion to other ECM proteins in addition to fibronectin. HEL cells were treated for 2 h with 20 μM IPA-3 and applied to wells coated with different ECM proteins or bovine serum albumin (BSA) as a control (Millicoat 96-well ECM screening kit, Millipore). After 1 h incubation at 37°C, culture medium with unattached cells was aspirated and the wells were washed once with Ca2+/Mg2+ containing buffer. The relative amount of attached cells was determined using calcein staining. Clear bars: untreated cells, dark bars: IPA-3-treated cells. The results are shown as means and standard deviations of sample quadruplicates.
(TIF)

Figure S6 **Detection of PAK1 and PAK2 expression using different anti-PAK antibodies in MOLM-7 cell lysate.**
(TIF)

Figure S7 **Expression levels of pPAK2 (Ser141) and PAK2 in cells treated with IPA-3.** Cells from different cell lines as indicated were treated for 2 h with IPA-3 at different concentrations: (1) control, (2) 2 μM, (3) 5 μM, (4) 10 μM and (5) 20 μM. Cells were lyzed and the protein expression levels were assessed by western-blotting. The band intensities for pPAK/actin and PAK2/actin are shown in Figure 6C of the paper.
(EPS)

Table S1 **Adhesivity of different cell lines to extracellular matrix proteins.**
(DOC)
