# Supplementary figures and images for: Group I PAK Inhibitor IPA-3 Induces Cell Death and Affects Cell Adhesivity to Fibronectin in Human Hematopoietic Cells
Source: PLoS One. 2014 Mar 24;9(3):e92560. doi: 10.1371/journal.pone.0092560 (PMC3963893; doi:10.1371/journal.pone.0092560)

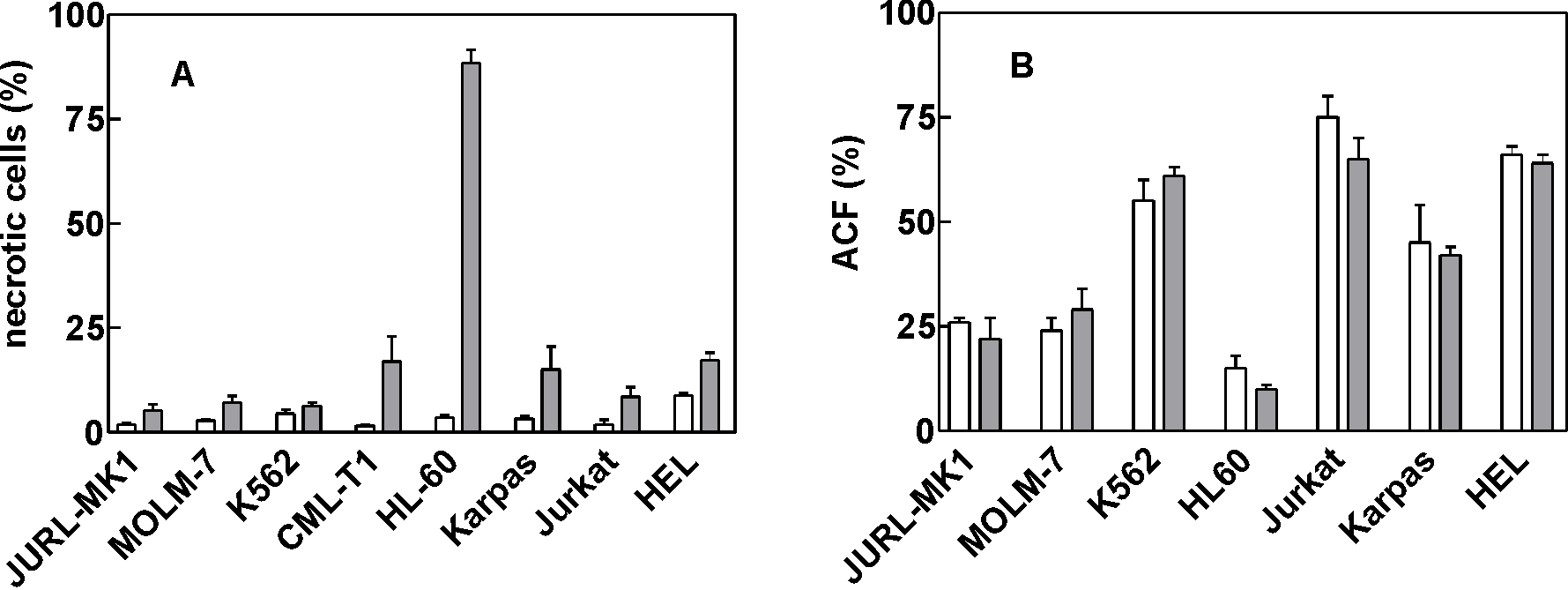

Supplement: Figure S2 — Effect of PIR3.5 on the viability and adhesivity of leukemic cell lines. Cells were treated with 20 μM PIR3.5 which is used as a control for IPA-3. White bars: controls (cells treated with DMSO), dark bars: cells treated with PIR3.5. A: effect of 48 h PIR3.5 treatment on the cell viability, means and s.d. from 3 independent experiments. B: effect of 2 h PIR3.5 treatment on the cell adhesivity to fibronectin, means and s.d. from sample quadruplicates. (TIF) [file pone.0092560.s002.tif]

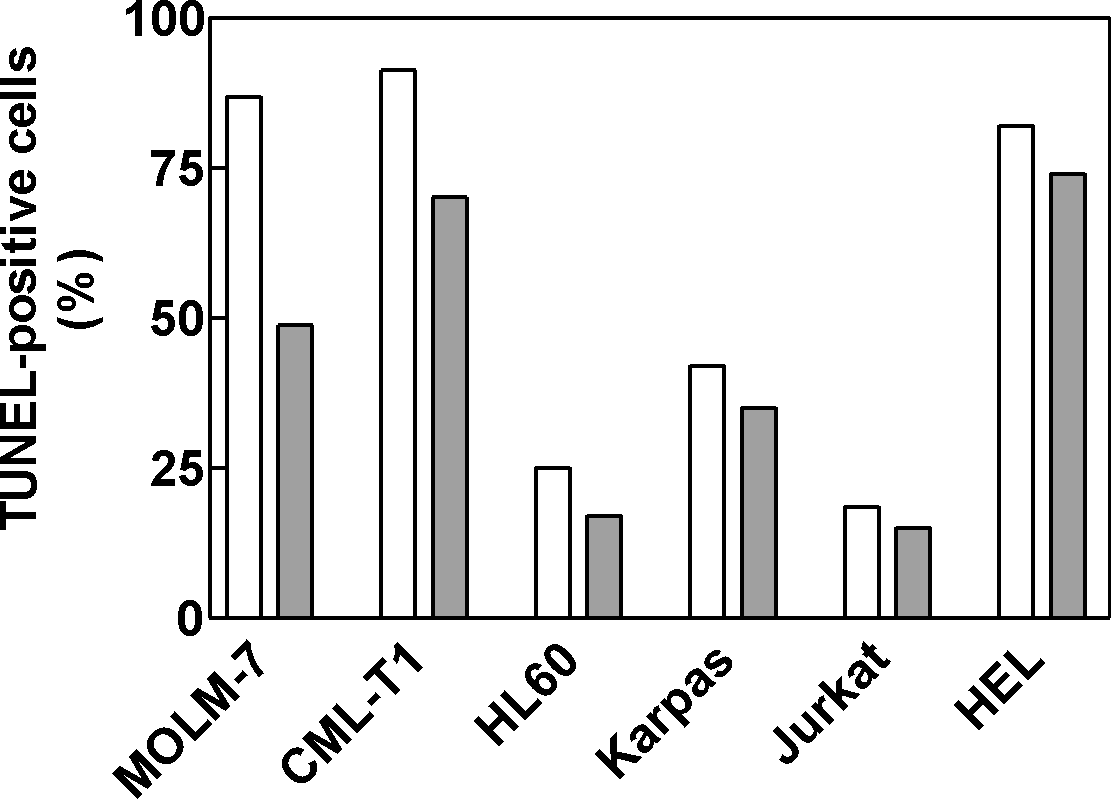

Supplement: Figure S3 — Effect of caspase inhibitor Q-VD-OPh on IPA-3-induced apoptotic DNA fragmentation. Cells were treated for 48 h with 20 μM IPA-3 alone (clear bars) or in combination with 20 μM Q-VD-OPh (dark bars) and apoptotic DNA breaks were detected using TUNEL assay. (TIF) [file pone.0092560.s003.tif]

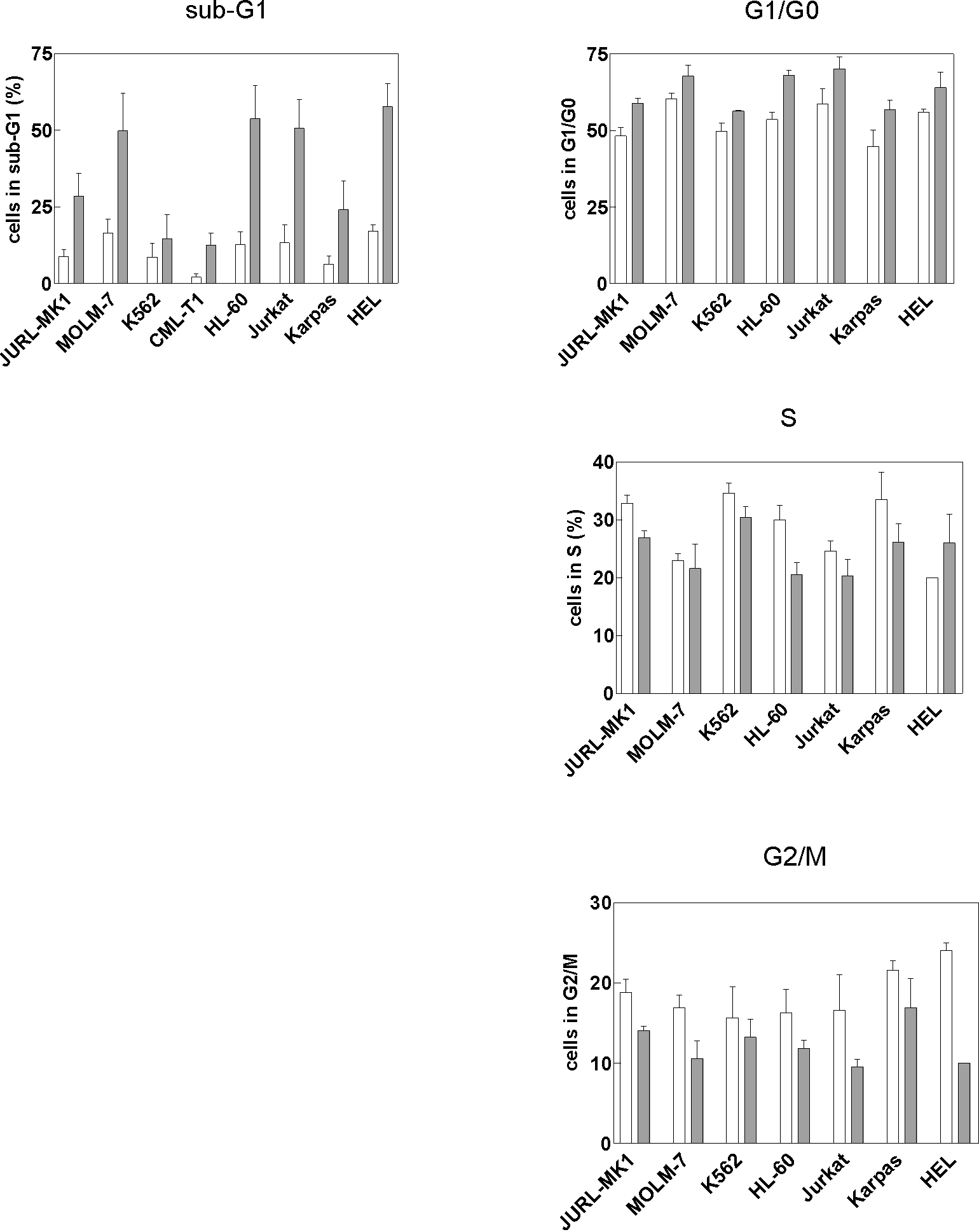

Supplement: Figure S4 — Cell cycle analysis of control and IPA-3-treated cell lines. Cells were treated with 20 μM IPA-3 for 24 h, the cell fraction in sub-G1 phase is shown on the left. The fraction of cells in G1/G0, S and G2/M phase are expressed as relative to G1/G0+S+G2/M (cells not in sub-G1) and shown on the right. CML-T1 cell line is mixed diploid/tetraploid and the cell cycle distribution thus cannot be derived from DNA content profiles. Means and s.d. from 3 independent experiments. White bars: controls, dark bars: IPA-3. (TIF) [file pone.0092560.s004.tif]

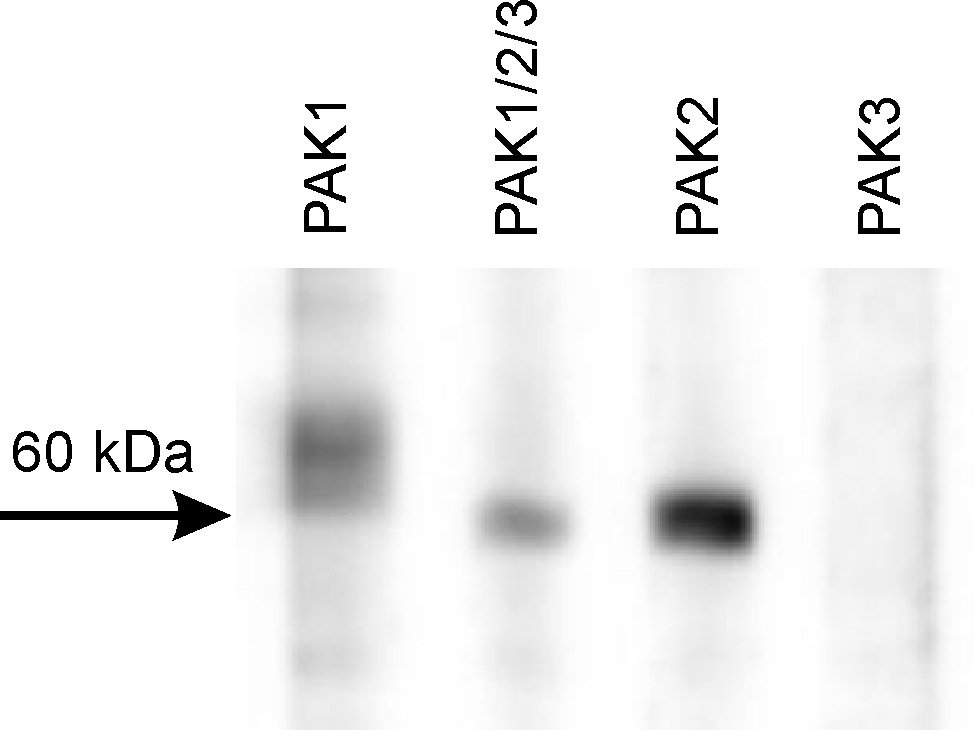

Supplement: Figure S6 — Detection of PAK1 and PAK2 expression using different anti-PAK antibodies in MOLM-7 cell lysate. (TIF) [file pone.0092560.s006.tif]
