# Supplementary material for: Group I PAK Inhibitor IPA-3 Induces Cell Death and Affects Cell Adhesivity to Fibronectin in Human Hematopoietic Cells
Source: PLoS One. 2014 Mar 24;9(3):e92560. doi: 10.1371/journal.pone.0092560 (PMC3963893; doi:10.1371/journal.pone.0092560)
Supplement: Table S1 — Adhesivity of different cell lines to extracellular matrix proteins. (DOC) [file pone.0092560.s008.doc]

Table S1: Adhesivity of different cell lines to extracellular matrix proteins.

Cell adhesivity to fibronectin (FN), vitronectin (VN), laminin (LAM), collagen I (COL I) and collagen IV (COL IV) was tested using Millicoat 96-well ECM screen kit (Millipore), according to the protocol described in Material and Methods section. The unspecific binding to BSA was less than 2%. Proteins binding less than 5% cells were considered non-interacting.

| **Cell line**: | **interacting ECM protein**: |
| --- | --- |
| JURL-MK1 | FN |
| MOLM-7 | FN |
| K562 | FN |
| CML-T1 | none |
| HL60 | FN |
| JURKAT | FN, LAM |
| Karpas-299 | FN, LAM |
| HEL | FN, VN, LAM, COL I, COL IV |
